# A Report of the Committee on the Abuse of Alcoholic Drinks from a Sanitary Standpoint

**Published:** 1896-04

**Authors:** 


### A Report of the Committee on the Abuse of Alcoholic Drinks from a Sanitary Standpoint.

This report was presented at the Denver meeting of the American Public Health Association by a committee previously appointed for the purpose. We are sorry to find the authors of this paper taking sides with the popular but deceptive and fallacious theory that the injury arising from alcoholic liquors is wholly due to their abuse, and that no reasonable objection can be offered against the moderate use of wine and beer. The astonishing position is taken that beer, claret and allied drinks aid digestion and assist in the conversion of starchy foods. Says the committee, "Wine strengthens and cheers without inebriating," an affirmation which to the physiologist is ludicrously absurd. Next month we shall undertake a somewhat extended review of this article, which, coming from so high an authority as the American Public Health Association, must have a more or less decided influence upon the public.—*Bulletin of Am. Med. Temp. Ass'n.*

